# Challenges in assessing ethical training in contemporary medicine: Psychometric evidence from two retention exams

**DOI:** 10.1016/j.clinsp.2026.101111

**Published:** 2026-09-06

**Authors:** Flavio Miura, Mauro Hilkner, Alfredo Inácio Fiorelli, Analía Arévalo, Angelo Vattimo, Christiane Cardoso Anicet Leite, Maria Alice Saccani Scardoelli, Mario Antonio Martinez Filho, Guilherme Lepski

**Affiliations:** a Hospital das Clínicas, Faculdade de Medicina, Universidade de São Paulo, São Paulo, SP, Brazil; b Conselho Regional de Medicina do Estado de São Paulo (CREMESP), São Paulo, SP, Brazil; c Laboratory of Medical Investigation LIM26, Faculdade de Medicina da Universidade de São Paulo, São Paulo, SP, Brazil; d Departments of Neurology, Psychiatry, and President of the Medical Ethics Committee, Hospital das Clínicas da Faculdade de Medicina da Universidade de São Paulo, São Paulo, SP, Brazil

**Keywords:** Ethics, Medical education, Psychometric assessment

## Abstract

**Objective:**

To evaluate the psychometric performance of two retention multiple-choice exams focused on practical ethics, deontology, and professional conduct administered to residents after interactive, in-person courses.

**Methods:**

We analyzed item difficulty (p), corrected point-biserial correlation (r_pb*), and internal consistency (KR-20) under Classical Test Theory. Thematic categories were assigned to each item based on course blueprints and question stems. Two cohorts (Exam I on June 4, 2025; Exam II on September 8, 2025) completed 20-item tests each. Analyses were performed in Excel with verification in Python.

**Results:**

Exam I showed KR-20 = 0.61 with a high overall proportion correct (mean p ≈ 0.94), limiting score variance and discrimination (mean r_pb*≈0.28). Exam II exhibited improved reliability (KR-20 = 0.83) and higher mean discrimination (mean r_pb*≈0.52). Although most items in both exams are considered to be easy, in the context of a retention test this pattern is also compatible with efficient incorporation of practical content after teaching, particularly in thematic domains more readily retained by residents. Ceiling effects therefore indicate not only reduced psychometric spread, but also probable high short-term retention in several practical topics; however, items with very high p values still have limited utility for distinguishing between stronger and weaker performers and for identifying content areas that may require reinforcement. Items framed as practical clinical or professional scenarios ‒ especially those involving autonomy and communication, documentation, civil/criminal liability, and relationships with health insurers ‒ showed the strongest discrimination. By contrast, digital ethics and advertising items were generally easy and less discriminative, possibly because these rules were already familiar, more directly memoizable, or presented in especially explicit formats during teaching.

**Conclusion:**

Psychometric monitoring of short retention exams in practical ethics and deontology supports continuous quality improvement and favors contextualized, case-based items that better differentiate residents in the domain of applicable professional rules and routine ethical-regulatory decisions. Such assessments should be a routine part of ethics curriculum development.

## Highlights

• Psychometric analysis improved ethics retention test performance.
• KR-20 increased from 0.61 to 0.83 after exam reformulation.
• Case-based items showed superior discrimination among residents.
• Practical ethics domains yielded the highest item discrimination.
• CTT metrics support continuous improvement of ethics education.

## Introduction

Contemporary medical practice is undergoing a structural transformation driven by the accelerated digitalization of healthcare, the expansion of telemedicine, the incorporation of artificial intelligence into diagnostic processes, and the growing presence of both physicians and patients in virtual environments and social media.^15^, ^16^, ^17^, ^18^ This transition redefines not only how care is delivered but also the practical conditions under which professional responsibility is exercised. In an increasingly connected world, the management of sensitive data, information security, public communication, and the relationship between clinical autonomy and institutional or automated protocols have become central challenges for daily medical practice and for safeguarding patients’ rights.

In this global context, new data protection frameworks ‒ such as the General Data Protection Regulation (GDPR) in the European Union, the Health Insurance Portability and Accountability Act (HIPAA) in the United States, and analogous regulations in Latin America and Asia ‒ impose rigorous standards for the confidentiality and traceability of medical information.^1^^,^^2^ At the same time, professional councils and international organizations, including the World Medical Association (WMA), continually revise their ethical guidelines, adapting traditional principles ‒ beneficence, non-maleficence, autonomy, and justice ‒ to interactions increasingly mediated by digital technologies and new organizational models of care.

Ethics education in medicine therefore includes at least two complementary domains. One domain concern broader moral deliberation in the face of ambiguity, conflict of values, and complex dilemmas. Another concerns the deontological and regulatory dimension of practice: the correct interpretation and application of professional duties, legal norms, institutional rules, and standards of conduct in everyday clinical life. The present educational intervention was intentionally centered on this second domain. In a large public academic hospital training approximately 800 residents per year, scalable assessment strategies are needed to verify whether trainees retained objective, applicable knowledge related to advertising, confidentiality, documentation, communication, relationships with health insurers, and other recurrent sources of ethical-regulatory exposure in real practice.

Accordingly, our aim was not to claim that short multiple-choice tests can capture the entirety of ethical competence. Rather, we examined whether such tests can function as practical retention assessments for a defined and educationally relevant component of ethics training: knowledge application in routine deontological decision-making. Under Classical Test Theory (CTT), three indicators are particularly informative for this purpose: the proportion correct (p), the corrected point-biserial correlation (r_pb*), and the Kuder-Richardson coefficient for dichotomous items (KR-20). Together, these indices provide complementary evidence of item difficulty, discrimination, and internal consistency, respectively.^3^, ^4^, ^5^, ^6^, ^7^ Prior research shows that contextualized items anchored in authentic professional situations can enhance discrimination and educational value compared with purely declarative items.^8^, ^9^, ^10^, ^11^ Building on this foundation, we analyzed two retention exams administered after interactive, in-person courses in medical ethics and bioethics to determine whether contextualized stems and blueprint refinement would improve measurement performance within this practical domain.

## Methods

Study design and setting: We conducted a psychometric analysis of two retention multiple-choice exams administered to medical residents at the end of interactive, in-person courses on ethics and bioethics, as part of a mandatory program in the first year of training. Residents are required to attend at least one of the courses. The educational objective of these exams was not to provide a comprehensive assessment of moral reasoning, but to verify short-term retention of practical and deontological content directly applicable to daily medical work. Exam I was administered on June 4, 2025, after a four-hour module that included talks on medical advertising, medical documentation, professional responsibility, and a simulated ethical judgment. Exam II took place on September 8, 2025, during a four-hour symposium including lectures on digital reputation and data protection, autonomy and communication, civil/criminal responsibility, relationships with health insurers, and a simulated ethical judgment. These exams were administered in two separate teaching activities and are therefore analyzed as two educational cohorts rather than as repeated measures of the same group. By design, Exam II placed greater emphasis on case-based learning than Exam I. Because the anonymized dataset was assembled for institutional educational monitoring, it did not retain participant-level variables allowing longitudinal linkage across exams or adjusted comparison by specialty, postgraduate year, or previous ethics exposure; this should be considered when interpreting between-exam differences and generalizability.

This observational educational study was reported in accordance with the STROBE (Strengthening the Reporting of Observational Studies in Epidemiology) Statement whenever applicable to psychometric and educational research.

The procedures followed the ethical regulations of the Clinics Hospital, Medical School, University of São Paulo, as well as national guidelines and legislation on research ethics. Participants were identified only by numeric codes and handled anonymously. This study analyzed anonymized educational assessment data obtained during institutional teaching activities. No personal, identifiable, or sensitive information was collected, recorded, or retained. According to Brazilian National Health Council Resolution n° 510/2016 (Article 1, sole paragraph, items VII and VIII), anonymous educational assessments and opinion surveys that do not involve identifiable human subjects are exempt from review by the CEP/CONEP system and do not require written informed consent. Therefore, formal Research Ethics Committee approval and protocol registration were not required.

All data necessary to interpret the results are presented within the manuscript and supplementary materials. Additional information can be provided upon direct request to the corresponding author.

Instruments: Each exam comprised 20 single-best-answer items (four options; one correct). Responses were coded as 1= correct and 0 = incorrect. The following indices were computed per item: p=A/N, where A is the number of correct responses and N is the total number of examinees; corrected point-biserial r_pb*=((X1−X0)/s_X)*sqrt(p*q), where X̄1 and X̄0 are mean total scores among examinees who answered the item correctly vs. incorrectly, s_X is the standard deviation of the total score, p is the proportion correct, and q = 1−p; and internal consistency via $KR-20=\left(k/(k-1)\right)*\left(1-\left(\Sigma_{p}\_iq\_i\right)/s\_X^{⋀}2\right)$.

Thematic mapping: Items were categorized into themes using the course blueprint and question stems: Medical Advertising and Digital Ethics; Medical Documentation and Reporting; Professional Responsibility and Ethical Procedures; Autonomy and Shared Decision-Making; Civil and Criminal Liability; Relationship with Health Insurers and Conflicts of Interest; Data Protection and Privacy (LGPD/GDPR); and Research Ethics. Analyses were performed in Microsoft Excel (2024) and validated in Python (pandas, numpy). This study was conducted in accordance with national regulations on research ethics. No personally identifiable data were analyzed.

The original spreadsheets with individualized responses were processed in Microsoft Excel (version 2024), with complementary Python scripts (pandas and numpy) used to automate analyses and cross-check calculations. The results were interpreted in light of classical psychometric literature,^3^, ^4^, ^5^, ^6^ with emphasis on the practical function of these exams as retention tools for objective, practice-oriented ethics content rather than as comprehensive measures of ethical competence.

## Results

A total of 410 residents (n1 = 410) answered the first examination, and 320 residents completed the second. Each question contained four alternatives with only one correct answer. Participant performance was coded in a binary format (1 = correct; 0 = incorrect), in accordance with Classical Test Theory (CTT). The two exams corresponded to separate educational cohorts, and the anonymized database did not allow resident-by-resident linkage between administrations.

Regarding examinee performance and reliability, Exam I exhibited high overall facility (mean p ≈ 0.94), constraining score variance and producing moderate internal consistency (KR-20 = 0.61). Exam II achieved markedly higher internal consistency (KR-20 = 0.83) and higher average discrimination. Although Exam II did not show a fully balanced distribution of item difficulty, the predominance of very easy items should be interpreted in light of the educational purpose of these instruments as retention tests. In this setting, very high p-values may reasonably reflect that core practical content was effectively incorporated after instruction, particularly in themes that residents encounter frequently in real clinical life. Ceiling effects therefore have a dual meaning here: from a psychometric standpoint, they reduce score dispersion and limit further differentiation; from an educational standpoint, they are compatible with high short-term retention and thus with effective teaching in specific domains. The psychometric gain in Exam II can therefore be understood not as a complete resolution of difficulty imbalance, but as an improvement in targeting and discrimination within a test that still captured strong retention of highly applicable content (Table 1).

**Table 1:** Classical test theory.

Item	Exam	p	Classification p	Corrected point-biserial (r_pb*)	Classification (r_pb*)	Theme
Q01	I	0.9684	Very easy	0.259	Moderate	Medical Advertising and Digital Ethics
Q02	I	0.9197	Very easy	0.2246	Moderate	Medical Advertising and Digital Ethics
Q03	I	0.9927	Very easy	0.4176	Excellent	Medical Advertising and Digital Ethics
Q04	I	0.6569	Moderate	0.0472	Low	Medical Advertising and Digital Ethics
Q05	I	0.9586	Very easy	0.2945	Moderate	Medical Advertising and Digital Ethics
Q06	I	0.7981	Easy	0.202	Moderate	Medical Documentation and Reporting
Q07	I	0.9976	Very easy	0.5955	Excellent	Medical Documentation and Reporting
Q08	I	0.9343	Very easy	0.2327	Moderate	Medical Documentation and Reporting
Q09	I	0.9878	Very easy	0.2734	Moderate	Medical Documentation and Reporting
Q10	I	0.9903	Very easy	0.2566	Moderate	Medical Documentation and Reporting
Q11	I	0.9562	Very easy	0.2773	Moderate	Professional Responsibility and Ethical Procedures
Q12	I	0.9489	Very easy	0.2882	Moderate	Professional Responsibility and Ethical Procedures
Q13	I	0.9976	Very easy	0.5955	Excellent	Professional Responsibility and Ethical Procedures
Q14	I	0.9927	Very easy	0.4775	Excellent	Professional Responsibility and Ethical Procedures
Q15	I	0.9903	Very easy	0.4288	Excellent	Professional Responsibility and Ethical Procedures
Q16	I	0.9854	Very easy	0.2338	Moderate	Professional Responsibility and Ethical Procedures
Q17	I	0.9878	Very easy	0.3662	Good	Professional Responsibility and Ethical Procedures
Q18	I	0.82	Easy	0.216	Moderate	Professional Responsibility and Ethical Procedures
Q19	I	0.9465	Very easy	0.2035	Moderate	Professional Responsibility and Ethical Procedures
Q20	I	0.9903	Very easy	0.3422	Good	Professional Responsibility and Ethical Procedures
Q01	II	0.9375	Very easy	0.4874	Excellent	Communication techniques in difficult scenarios
Q02	II	0.9625	Very easy	0.5029	Excellent	Communication techniques in difficult scenarios
Q03	II	0.925	Very easy	0.3858	Good	Communication techniques in difficult scenarios
Q04	II	0.9562	Very easy	0.2364	Moderate	Civil and Criminal Liability
Q05	II	0.9562	Very easy	0.2364	Moderate	Civil and Criminal Liability
Q06	II	0.9812	Very easy	0.541	Excellent	Civil and Criminal Liability
Q07	II	0.7969	Easy	0.1257	Low	Civil and Criminal Liability
Q08	II	0.9781	Very easy	0.6517	Excellent	Civil and Criminal Liability
Q09	II	0.9219	Very easy	0.3877	Good	Autonomy and Shared Decision-Making
Q10	II	0.9875	Very easy	0.4649	Excellent	Autonomy and Shared Decision-Making
Q11	II	0.9906	Very easy	0.6414	Excellent	Autonomy and Shared Decision-Making
Q12	II	0.9719	Very easy	0.429	Excellent	Autonomy and Shared Decision-Making
Q13	II	0.9688	Very easy	0.3997	Good	Autonomy and Shared Decision-Making
Q14	II	0.9344	Very easy	0.3446	Good	Autonomy and Shared Decision-Making
Q15	II	0.9875	Very easy	0.7993	Excellent	Autonomy and Shared Decision-Making
Q16	II	0.9812	Very easy	0.4241	Excellent	Relationship with Health Insurers and Conflicts of Interest
Q17	II	0.9906	Very easy	0.7511	Excellent	Relationship with Health Insurers and Conflicts of Interest
Q18	II	0.95	Very easy	0.4217	Excellent	Ethics in Research
Q19	II	0.9781	Very easy	0.6891	Excellent	Medical Confidentiality
Q20	II	0.9844	Very easy	0.6753	Excellent	Medical Confidentiality

With respect to item difficulty and discrimination, in Exam I, the average r_pb* was 0.28, with few items exceeding 0.40. In Exam II, the mean r_pb* increased to 0.52, with several items reaching 0.60‒0.80 despite the persistence of very high p values. This pattern suggests that scenario-based items can better separate examinees even in relatively easy examinations. The key practical message is that strong discrimination does not fully compensate for excessive item easiness. Items answered correctly by nearly all examinees provide little information about differences in the strength of learning, even when their point-biserial coefficients are numerically high. In particular, once p exceeds approximately 0.95, an item becomes less useful for distinguishing robust retention from responses based on obvious cues, simple recognition, or occasional guessing. Future examinations should therefore preserve the contextualized, case-based format while also including a greater proportion of moderately difficult items.

**Fig. 1:**
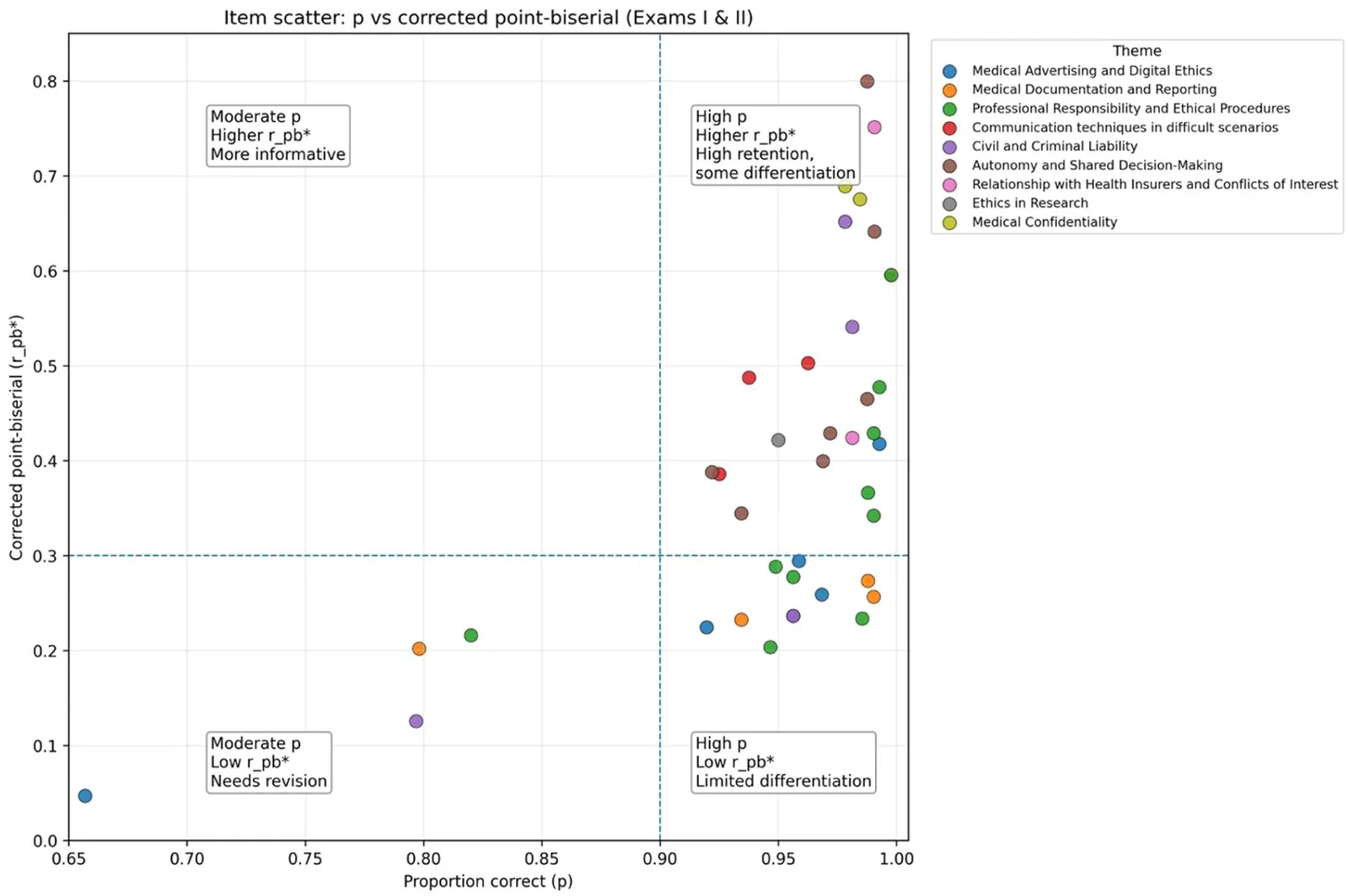
Item scatter: p vs corrected point-biserial (Exams I & II). Each point represents a test item. The x-axis shows the proportion correct (p), and the y-axis shows the corrected point-biserial (r_pb*), an index of item discrimination. Colors indicate thematic categories, using a higher-contrast palette for readability. Dashed reference lines and quadrant labels help distinguish items with limited differentiation at high p from those offering better informational value at moderate p and higher r_pb*.

**Fig. 2:**
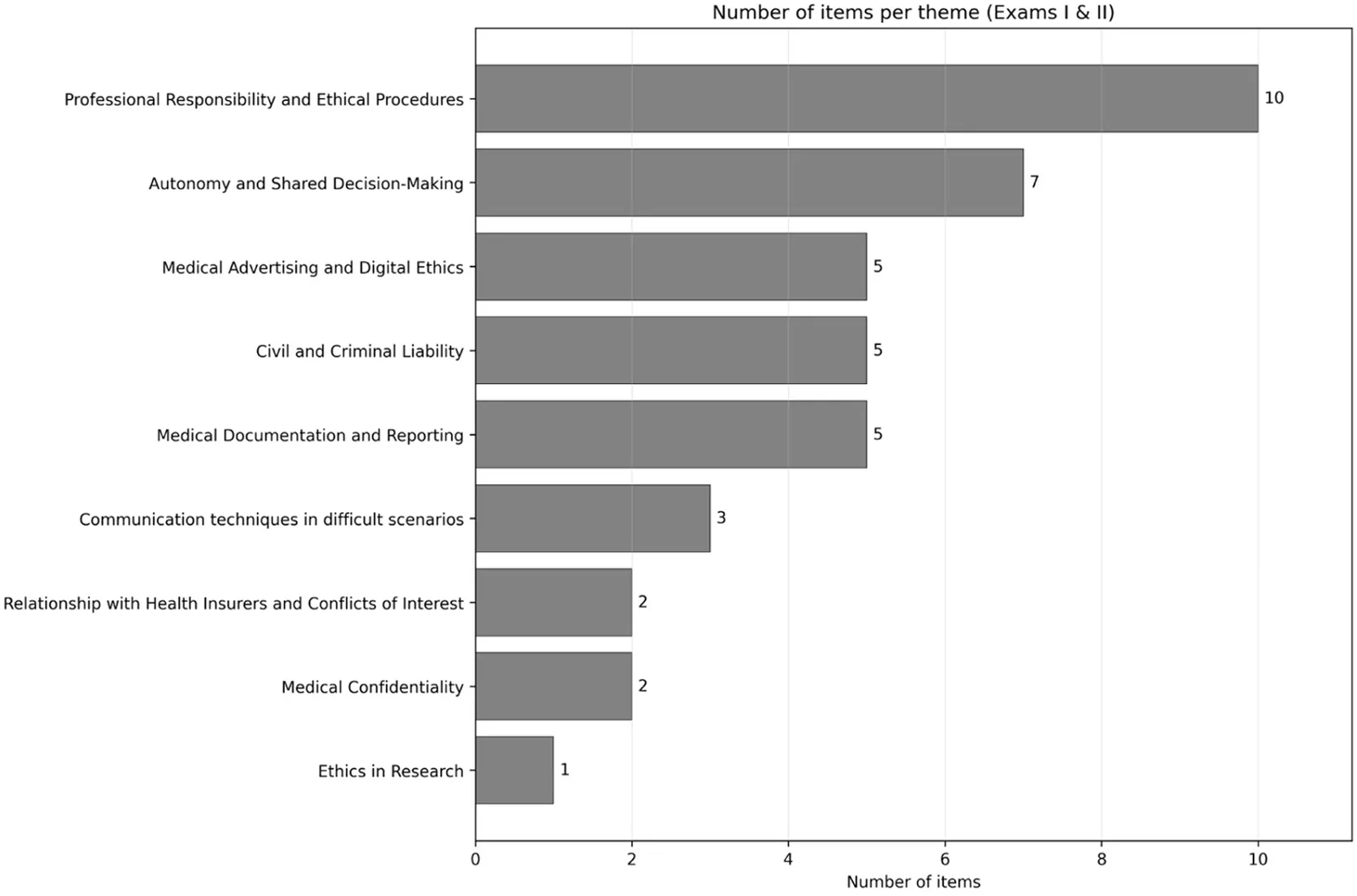
Number of items per theme (Exams I & II). Horizontal bars indicate the count of items within each thematic category, and labels at the bar ends show absolute frequencies. This distribution highlights content balance across the blueprint and helps identify areas that may benefit from calibration in future assessments.

From a thematic perspective, items on medical documentation, civil responsibility, autonomy and communication, and relationships with health insurers showed the most useful discriminative behavior, likely because they required interpretation of practical rules in recognizable professional scenarios rather than mere literal recall. These categories included questions such as under what circumstances treatments are considered experimental and when third parties may be granted access to patients’ medical records. This finding is consistent with the educational design of the course, which emphasized the application of deontological norms to routine medical situations. At the same time, the very high facility observed in some thematic blocks may indicate that certain contents were more readily incorporated by residents, possibly because they were presented in a concrete and directly applicable format. By contrast, items on digital advertising rules and some procedural topics were predominantly very easy and therefore contributed less to score differentiation, suggesting either higher retention of these contents, greater prior familiarity through institutional and social-media exposure, or insufficient challenge in item construction; future iterations should therefore increase scenario complexity while preserving practical relevance (Fig. 1, Fig. 2).

## Discussion

This study indicates that contextualized stems and blueprint refinement can improve the psychometric quality of short retention assessments in practical ethics and professional conduct. The contribution of the study is not the discovery of new psychometric principles, but the demonstration of how established principles behave in a large-scale, real-world educational setting focused on deontological and regulatory content that physicians must apply in routine practice. In this context, objective testing serves a specific and legitimate purpose: not to exhaustively measure ethical competence, but to verify whether trainees retained essential knowledge needed to avoid recurrent professional errors involving documentation, confidentiality, communication, advertising, civil exposure, and institutional relationships. At the same time, our educational interpretation of high p values should remain cautious. In retention testing, a predominance of correct answers is compatible with successful short-term incorporation of core content; however, once items become extremely easy, they also lose power to distinguish residents who have more firmly internalized the material from those responding by simple recognition of explicit rules or, less commonly, guessing.

In practical terms, ethics curricula should intentionally couple interactive learning (e.g., simulated ethical judgments and case deliberations) with psychometrically monitored assessments tailored to clearly defined learning goals. For large residency programs, short objective tests remain operationally important when the educational target is retention of applicable norms and professional rules. Routine reporting of p, r_pb*, and KR-20 at the item and test levels enables iterative refinement, supports defensibility, and helps identify content domains in which residents may remain vulnerable to ethical-regulatory problems in daily practice. While broader competency-based methods are indispensable for evaluating complex moral reasoning and behavior, they are complementary to ‒ not replacements for ‒ scalable retention assessments of deontological knowledge. The present findings should nevertheless be interpreted in light of cohort limitations: the two exams were applied to separate groups of residents in the first year of training, as part of a mandatory program in Ethics, within the same institutional setting, and the anonymized dataset did not preserve background variables that would allow stronger comparison of specialty mix, training level, or prior ethics exposure. Future work may therefore combine this approach with other modalities, including observed performance, written justifications, or programmatic assessment frameworks.^10^, ^11^, ^12^, ^13^, ^14^ In ethics training, such a framework could integrate multiple low-stakes data points collected longitudinally, including short knowledge tests, written analyses of clinical dilemmas, performance in simulated ethical hearings, multisource feedback, reflective narratives, and direct observation of communication and documentation practices. Rather than relying on a single examination, faculty committees could periodically synthesize this information to provide individualized feedback, identify persistent areas of vulnerability, and guide remediation or progressive entrustment in ethically sensitive professional activities. The psychometric monitoring described in this study could thus constitute one component of a broader assessment system in which decisions are based on the aggregation of complementary sources of evidence, consistent with principles of validity, fairness, and generalizability.

## Conclusion

Psychometric supervision of short retention assessments ‒ by monitoring p, r_pb*, and KR-20 ‒ helps align measurement with educational intent and improves sensitivity to differences in performance within a defined domain of practical ethics. Our results show that contextualized, case-based items can yield superior discrimination compared with purely declarative recall. They also suggest that, in a retention-testing scenario, very high proportions of correct answers may partly represent successful teaching and strong short-term incorporation of practical ethical-regulatory content, even though further calibration remains desirable to reduce ceiling effects and expand score variance. In an era defined by digital medicine, data protection, telemedicine, and increased regulatory exposure, ethics programs should combine interactive teaching with psychometrically guided assessment cycles to strengthen practical knowledge, accountability, and safer professional conduct.

## Declaration of competing interest

The authors declare no conflicts of interest.

## Data Availability

The datasets generated and/or analyzed during the current study are available from the corresponding author upon reasonable request.

## References

[1] European Parliament, Council of the European Union (2016). Regulation (EU) 2016/679 of the European Parliament and of the Council of 27 April 2016 on the protection of natural persons with regard to the processing of personal data and on the free movement of such data, and repealing Directive 95/46/EC (General Data Protection Regulation). Off J Eur Union.

[2] U.S. Congress (1996). Health insurance portability and accountability act of 1996 (HIPAA). Pub L.

[3] Kelley T.L. (1939). The selection of upper and lower groups for the validation of test items. J Educ Psychol.

[4] Nunnally J.C., Bernstein I.H. (1994). Psychometric Theory.

[5] Crocker L., Algina J. (2006). Introduction to classical and modern test theory.

[6] Lord F.M., Novick M.R. (1968). Statistical Theories of Mental Test Scores.

[7] Kuder G.F, Richardson M.W. (1937). The theory of the estimation of test reliability. Psychometrika.

[8] Downing S.M., Downing S.M., Yudkowsky R (2009). Assessment in Health Professions Education.

[9] Haladyna T.M, Downing S.M, Rodriguez M.C. (2002). A review of multiple-choice item-writing guidelines for classroom assessment. Appl Meas Educ.

[10] Schuwirth L.WT., van der Vleuten C.PM. (2011). Programmatic assessment: from assessment of learning to assessment for learning. Med Teach.

[11] Norcini J., Anderson B., Bollela V., Burch V., Costa M.J, Duvivier R. (2018). 2018 Consensus framework for good assessment. Med Teach.

[12] Kane M.T. (2013). Validating the interpretations and uses of test scores. J Educ Meas.

[13] American Educational Research Association, American Psychological Association, National Council on Measurement in Education (2014). Standards for Educational and Psychological Testing.

[14] Brennan R.L. (2001).

[15] Chretien K.C, Kind T. (2013). Social media and clinical care: ethical, professional, and social implications. Circulation.

[16] Ventola C.L. (2014). Social media and health care professionals: benefits, risks, and best practices. P T.

[17] van der Boon R.MA., van der Zanden V., van de Watering M., van Delden J.JM., Bredenoord A.L. (2024). Risks and benefits of sharing patient information on social media: a digital dilemma. Front Digit Health.

[18] Giuffrida A., Koutrakis A., Fiester A., Barry J., Moyer K.J, Brinster C.J. (2024). Social media behavior guidelines for healthcare professionals: an American perspective. J Multidiscip Healthc.

